# Ultrasmall all-optical plasmonic switch and its application to superresolution imaging

**DOI:** 10.1038/srep24293

**Published:** 2016-04-11

**Authors:** Hsueh-Yu Wu, Yen-Ta Huang, Po-Ting Shen, Hsuan Lee, Ryosuke Oketani, Yasuo Yonemaru, Masahito Yamanaka, Satoru Shoji, Kung-Hsuan Lin, Chih-Wei Chang, Satoshi Kawata, Katsumasa Fujita, Shi-Wei Chu

**Affiliations:** 1 Department of Physics, National Taiwan University, No. 1, Sec. 4, Roosevelt Rd., Taipei 10617, Taiwan; 2 Department of Applied Physics, Osaka University, 2-1 Yamadaoka, Suita, Osaka 565-0851, Japan; 3 Institute of Physics, Academia Sinica, 128 Sec. 2, Academia Rd., Nankang, Taipei 11529, Taiwan; 4 Center for Condensed Matter Sciences, National Taiwan University, No. 1, Sec. 4, Roosevelt Rd., Taipei 10617, Taiwan; 5 Molecular Imaging Center, National Taiwan University, No. 1, Sec. 4, Roosevelt Rd., Taipei 10617, Taiwan

## Abstract

Because of their exceptional local-field enhancement and ultrasmall mode volume, plasmonic components can integrate photonics and electronics at nanoscale, and active control of plasmons is the key. However, all-optical modulation of plasmonic response with nanometer mode volume and unity modulation depth is still lacking. Here we show that scattering from a plasmonic nanoparticle, whose volume is smaller than 0.001 μm^3^, can be optically switched off with less than 100 μW power. Over 80% modulation depth is observed, and shows no degradation after repetitive switching. The spectral bandwidth approaches 100 nm. The underlying mechanism is suggested to be photothermal effects, and the effective single-particle nonlinearity reaches nearly 10^−9^ m^2^/W, which is to our knowledge the largest record of metallic materials to date. As a novel application, the non-bleaching and unlimitedly switchable scattering is used to enhance optical resolution to λ/5 (λ/9 after deconvolution), with 100-fold less intensity requirement compared to similar superresolution techniques. Our work not only opens up a new field of ultrasmall all-optical control based on scattering from a single nanoparticle, but also facilitates superresolution imaging for long-term observation.

In the last century, one of the most significant technical revolutions is the rapid development of electronics, whose driving engines are those nonlinear and active components, including diodes and transistors. The major issue that slows down recent development of electronic circuits is the loss at interconnects. Photonic circuits can provide less losses with higher densities of information^1^. To advance electronic circuit into photonic circuit, active components such as all-optical switches are indispensible. An ideal all-optical switch should fulfill several important requirements: (i) as small as nanoscale in size; (ii) high speed; (iii) large switching modulation depth; (iv) low-power request; (v) broadband operation. Recently, various schemes of all-optical switches have been proposed/demonstrated. The majority of them relies on cavity effects, such as photonic crystal nanocavities^2^, silicon-based microring resonator^3^, individual semiconductor nanowires^4^, and quantum Zeno effect^5^. The advantages of using cavities are outstanding switching modulation depth with reduced energy request. Single-photon switch has been achieved by coupling photonic crystal cavities with a quantum dot^6^,^7^. The capability of cascading and multiple fan-out has been demonstrated^8^. However, due to the existence of cavities, the switch size cannot meet the demand of nanoscale. The quantum Zeno switch requires bulky optical elements. The microring resonator is much smaller, but the diameter of the ring is on the order of 10 μm. Although the modal volume of a photonic crystal switch is less than 0.1 μm^3^, when considering the size of the surrounding crystal structures, the real volume of the switch is much larger. A single nanowire is much smaller than the whole photonic crystal structure^4^, but the volume of the nanowire still approaches 1 μm^3^. In addition, due to the existence of cavity, the response time is longer than tens of picoseconds, and the applicable optical bandwidth is limited to only a few nanometers.

There are also cavity-free systems showing all-optical switch capabilities, for example, by utilizing multiexcitons in semiconductor nanocrystals^9^, atomic Rydberg states^10^,^11^, or even a single dye molecule^12^. Their active sites are all very small in volume. However, not only the switching modulation depths are typically as low as a few percent, and the switching response time are longer than nanosecond, but also the atomic/molecular switches have to be kept at ultralow temperature (1.4 K), not practical for realistic applications.

Plasmonics provide the possibility to shrink the size of optical components into sub-wavelength regime, and act as the bridge between electronics and photonics^13^. All-optical active control of plasmonic response provides the potential of ultrafast speed and ultrasmall volume of interaction^14^. All-optical switches based on plasmonics have also been demonstrated recently^15^,^16^,^17^,^18^,^19^,^20^,^21^. It is known that the major obstacle of active plasmonics is the intrinsically weak optical nonlinearities of surface plasmon, and thus most of the previous demonstrations rely on combination of molecular nonlinearity with surface plasmons^15^,^16^,^17^,^18^,^19^, while few utilizes the intrinsic photon-plasmon interaction^20^,^21^. Except the one with liquid crystal, all plasmonic switches exhibit the advantage of ultrafast response, at least on the scale of picosecond. However, the switching modulation depths are mostly as low as less than 10% in these works, and most of them require long waveguide or periodic structures. Furthermore, for those combined with organic molecules, they are not compatible with semiconductor fabrication process.

In plasmonic particles, it is well known that scattering can be extraordinarily strong near resonance, and the scattering intensity/spectrum is very sensitive to local conditions. The scattering cross section of a spherical nanoparticle is known as


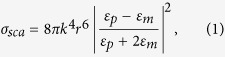


where *k* is the wave vector, *r* is the radius of particle, *ε*_*p*_ is the dielectric constant of the particle, and *ε*_*m*_ is the dielectric constant of the surrounding medium. By coupling photons into plasmons, the dielectric constant of the particle can be considerably modified, thus significantly changing the scattering efficiency. Here we demonstrate an all-optical plasmonic switch based on active control of scattering within a single gold nanoparticle, whose mode volume is less than 0.001 μm^3^. The modulation depth of this ultrasmall switch reaches 80%, and the switching behavior is active within the broad plasmonic resonance band. Combined with a setup similar to stimulated emission depletion (STED) microscope, our study demonstrated the first resolution enhancement beyond the diffraction limit based on all-optical switch of scattering.

## Results

### All-optical switching of plasmonic scattering

**Figure 1:**
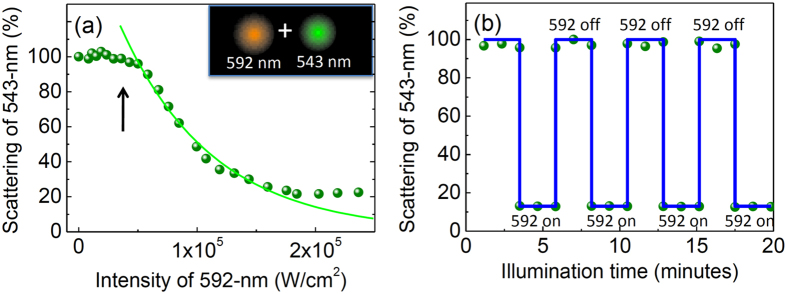
All-optical switching of scattering in a single gold nanoparticle. (**a**) The scattering intensity (normalized to the incident probe beam at λ = 543 nm with fixed intensity = 30 W/cm^2^) of an individual gold nanosphere as a function of the intensity of a control beam at λ = 592 nm. When the intensity of the control beam increases beyond 6 × 10^4^ W/cm^2^, the scattering at λ = 543 nm starts to decrease. Green line is fitting for exponential decay with a threshold. (**b**) Demonstration of reversible switching of scattering for prolonged observation without bleaching (data were truncated for longer illumination time). Here the intensity of the λ = 592 nm control beam is 2 × 10^5^ W/cm^2^.

With the aid of a reflection confocal microscope equipped with multiple continuous laser wavelengths, we are able to measure backward scattering of one wavelength from a single gold nanoparticle, while gradually increasing the intensity of another wavelength that is also focused onto the same nanoparticle to observe the all-optical switching behavior. Here we use gold nanoparticles with 80-nm diameter, so the plasmon resonance peak is around 590 nm. In Fig. 1(a), the control laser is at λ = 592 nm, which is very close to the peak, and the probe laser is at λ = 543 nm, which locates at the edge of the surface plasmon resonance (SPR) band. The focal spots of the two wavelengths are overlapped onto a single gold nanoparticle, but only the scattering of λ = 543 nm is recorded. The intensity of 543-nm beam is fixed at 30 W/cm^2^, which is not adequate to induce any nonlinearity, and the intensity of the 592-nm beam gradually increases. When the intensity of the 592-nm laser is low (<5 × 10^4^ W/cm^2^), the scattering intensity of 543-nm keeps constant, i.e. not dependent on 592-nm.

The most intrigue finding is that when the intensity of 592-nm beam increases into the nonlinear regime, the scattering at 543 nm begins to decrease. Remarkably, when the excitation intensity at 592 nm reaches 2 × 10^5^ W/cm^2^, more than 80% of the scattering at 543 nm is suppressed, demonstrating the all-optical switching behavior. The corresponding average power is less than 100 μW, and the energy absorbed by the nanoparticle is less than the requirement to write a bit in a DVD. Comparing to previous observations of nonlinear responses in plasmonic materials with pulsed lasers^22^,^23^, where 10^10^ W/cm^2^ intensity is used, in our results, not only the required excitation intensities for scattering switching is reduced by several orders of magnitudes, but the modulation depth of switch is much larger. The reason of less nonlinearity with pulsed excitation is that, for picosecond dynamics of gold, hot electron dominates the response. Nevertheless, here we use continuous-wave lasers, so the effect of hot lattice may play a major role in the significantly enhanced nonlinearity. More discussion regarding to the mechanism of all-optical switch will be given in the next section.

The repeatability and long-term stability of the all-optical switch are demonstrated in Fig. 1(b). No signal quenching is observed during prolonged and repeated on/off switch, showing its great potential for demanding industrial applications. In addition, the perfect long-term stability, lasting for hours with negligible switch degradation, excludes irreversible thermal effects, such as melting, to be likely mechanisms for our observation^24^.

### Mechanism of the all-optical switching

There are several possible mechanisms of the single-particle nonlinear switch, including contributions from intraband transition, interband transition, hot electron, and hot lattice. The former three factors have been extensively considered in the late 80’s^25^ when studying the nonlinear response of gold, and the conclusion is that hot electron contribution is more significant than the intraband or interband transitions, which are respectively dominating in the long and short wavelengths, even at thermal equilibrium. However, in these early works, the contribution of lattice is completely ignored, largely because femtosecond excitations were adopted.

**Figure 2:**
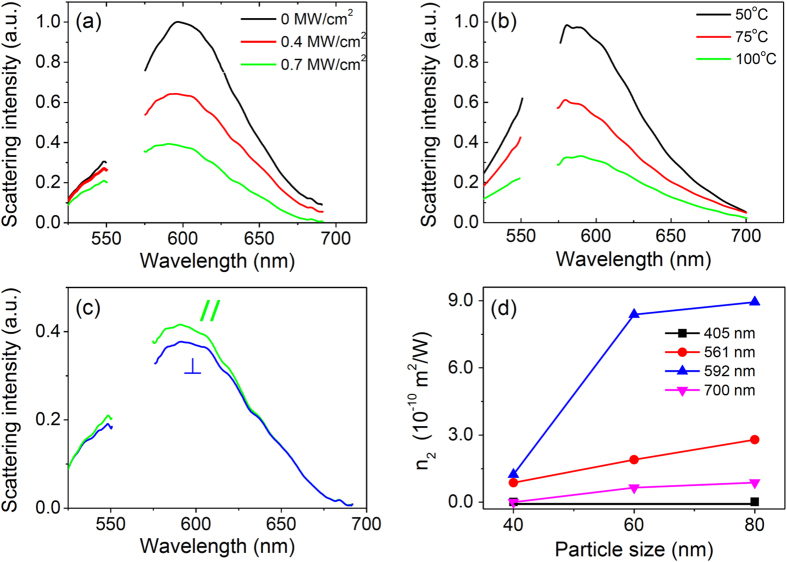
Wavelength, thermal, polarization, and size dependencies of scattering switching. (**a**) By collinearly illuminating a 561-nm laser and a supercontinuum laser onto the sample, scattering spectra with different 561-nm intensities are obtained. In the measurement, a notch filter for 561-nm is inserted, so there is no data points around that spectral region. (**b**) By heating up the nanoparticles, scattering from a single particle drops significantly, revealing the underlying mechanism is thermally induced change of dielectric constant. (**c**) Almost identical scattering spectra are obtained when the 561-nm laser polarization is either perpendicular or parallel to the supercontinuum polarization, manifesting that the switching effect is polarization independent with the nanosphere. (**d**) The nonlinear index distribution with different particle sizes and excitation wavelengths.

In order to identify the major mechanism, we first carried out spectral characterization. To allow observation of SPR peak position during spectral measurement, a 561-nm laser was used as the pump, and the broadband scattering was probed by a supercontinuum laser, along with a notch filter to block 561-nm in front of the spectrometer. From Fig. 2(a), scattering across the whole SPR spectral range can be simultaneous suppressed by a single pump wavelength, excluding the possibility of intraband or interband transition to be the dominating factor. In addition, this result manifests the feasibility of broadband all-optical switch operation, as long as the control and probe wavelengths are located within the SPR band, whose bandwidth is typically more than 100-nm.

To further confirm the mechanism to be the photothermal effect, we heat up the nanoparticle sample with a hot plate directly, and monitor the scattering spectrum variation of a single particle, as presented in Fig. 2(b). As a result, significant single-particle scattering intensity reduction with increasing temperatures is observed. A very nice correspondence is found between Fig. 2(a,b), showing that not only the mechanism of all-optical switch is well addressed, but also the potential of this effect as a sensitive nanometric thermometer. The slight blue shift of the SPR peak in Fig. 2(b) may be the result of size variation of the nanoparticles. More than 70% suppression ratio can be achieved with less than 100-degree temperature variation, accounting for the full reversibility in Fig. 1(b).

Following this mechanism, it is natural to expect that for spherical nanoparticles, polarization should not affect the switch performance. As demonstrated in Fig. 2(c), the switching modulation depth remains the same no matter the pump polarization is perpendicular or parallel to the polarization of supercontinuum.

The result supports once again the dominating factor of photothermal effect, and excludes the role of instantaneous nonlinear polarization due to intraband/interband transition. In view of applications, the polarization insensitivity enables arbitrary choice of polarizations for signal and control beams, very beneficial for signal processing and identification. Of course, if an asymmetric geometry, such as nanorods, is adopted, an additional polarization control can be incorporated, adding a new dimension to the all-optical switch.

The next issue is to distinguish between hot electron and hot lattice. In our experiments of Fig. 2(a,b), where continuous-wave lasers and hot plates are used, both electrons and lattices are heated up simultaneously. It is known that by adopting an ultrafast laser, whose peak power is very high but the average power is relatively low, the temperature of electrons can be raised to more than thousand degrees within a very short period of time, while the temperature of lattice remains nearly unchanged, even when energy has been fully relaxed to phonons^26^. However, from early experiments of ultrafast all-optical switch based on absorption from an ensemble of nanoparticles, the modulation depth is only about 10%, at the peak intensity level of 10^10^ W/cm^2^. The corresponding nonlinearity is induced by hot electron effect, and is much smaller than what we have found here (>80% modulation at 10^5^ W/cm^2^). Therefore, in our continuous-wave experiment, the nonlinearity is more likely to be the result of hot lattice, as we will address in more details below.

It is well known that hot electron contribution induces the Fermi smearing effect, which is not a coherent process, with turn-on and relaxation time of 0.5 ps and a few ps, respectively^27^. Thus the thermal effect accumulates with long pulses. For example, when the excitation pulse width is less than 1 ps, the equivalent nonlinear refractive index of bulk gold is on the order of 10^−15^ m^2^/W 28. When the pulse become 30 ps long, the nonlinear index rises to 10^−13^ m^2^/W. However, for pulses as long as 0.71 ns, which is much larger than the hot electron lifetime of gold, the nonlinearity increases to 10^−12^ m^2^/W. It is suggested that this large nonlinearity is the result of sample heating, i.e. hot lattice^28^, not hot electron. Please note that the above numbers are based on bulk measurement. In the case of nanoparticles, the nonlinearity would be further enhanced by the local field enhancement factor^29^, as well as by the reduced thermal conductivity between the gold nanoparticles and surrounding medium due to the coated surfactants^30^. By using a CW laser and immersing particles in oil, a recent study indicates that hot-lattice nonlinearity of gold nanoparticles reaches 1.9 × 10^−11^ m^2^/W, even when the excitation wavelength is not at the top of SPR peak^30^.

By deriving the nonlinear index based on our recent approaches^31^ (see Method for derivation), Fig. 2(d) shows the wavelength and size dependencies of nonlinear indices. Note that when the wavelength is around the peak of SPR, the nonlinearity can be as high as 8.8 × 10^−10^ m^2^/W. To our knowledge, this is the largest nonlinear index ever reported with gold nanostructures, and is much larger than the possible contribution from hot electron effects. In Fig. 2(d), when the excitation wavelength is slightly off resonance, the magnitude of nonlinear index becomes similar to the recent z-scan study^30^. Although ref. 30 measures nonlinear absorption from an ensemble of nanoparticles, and our work detects nonlinear scattering from a single nanoparticle, the similar magnitude of nonlinear index indicates similar underlying mechanism. Therefore, we conclude that the mechanism of our all-optical switch to be hot lattice effect.

With the hot lattice mechanism, the nonlinear index should be very sensitive to both particle sizes and excitation wavelengths, which affect the amount of energy that is absorbed by the particle, as shown in Fig. 2(d). Apparently, the nonlinear index grows higher with large particles. Since the absorption cross-section increases with the volume of nanoparticle, while heat dissipation is proportional to the surface area of the particle, it is reasonable to expect higher temperature for larger particles at equilibrium^32^,^33^. On the other hand, when the excitation wavelength is closer to the peak of SPR band, where the absorption is greatly enhanced, the nonlinear index is larger.

### Resolution enhancement based on all-optical switching of scattering

From the above results, the newly discovered switchable scattering is ready to improve the microscopic spatial resolution via STED-like techniques. STED relies on all-optical switch of fluorescent molecules, which exhibits limitations of molecular stability, switching reversibility, and photobleaching. With the exceptional stability of scattering-based all-optical switch, here we demonstrate suppression of scattering imaging (SUSI) that can significantly improve spatial resolution for plasmonic nanostructures.

**Figure 3:**
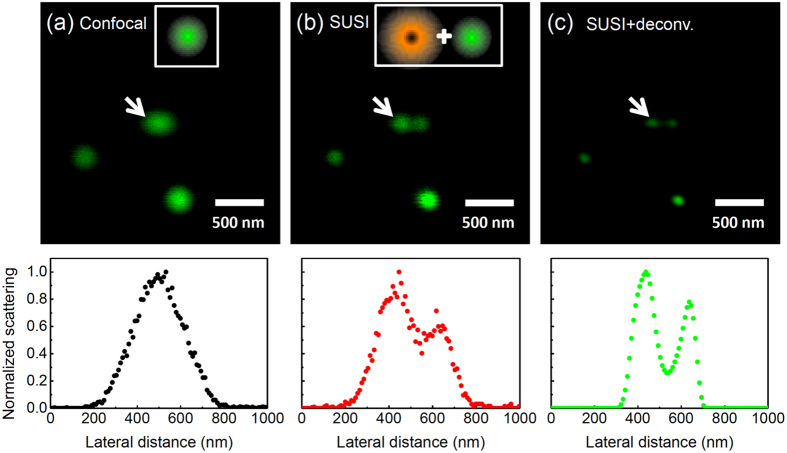
Resolution enhancement based on a STED-like setup and all-optical switching of scattering from isolated gold nanoparticles. (**a**) The reflection confocal image collecting the scattering of λ = 543 nm excitation. (b) Resolution enhancement of SUSI when incorporating a donut-shaped beam at λ = 592 nm. Clearly the point spread functions of all objects are reduced when the λ = 592 nm beam (intensity = 2 × 10^5^ W/cm^2^) is on. The arrows denote two gold nanospheres that are not resolvable in (**a**) but can be identified in (**b**). The imaging speed is ~1 second/frame. (**c**) After deconvolution of the SUSI image shown in (**b**), the gold nanospheres are clearly identified with resolution approaching λ/9.

Figure 3(a) shows the back-scattered confocal image of 80-nm gold nanospheres under single excitation at λ = 543 nm. The full width at half maximum (FWHM) of a single gold nanosphere is 180 nm, corresponding to the expected confocal resolution. In Fig. 3(b), the λ = 592 nm beam is converted into a donut shape by a vortex phase plate, and the central hole is overlapped with the λ = 543 nm excitation, similar to STED implementation^34^. When we increase the λ = 592 nm laser intensity to 2 × 10^5^ W/cm^2^, significant improvements of spatial resolution are obtained. As shown in Fig. 3(b), two adjacent particles that cannot be resolved by confocal imaging can now be clearly distinguished by SUSI. Note that the FWHMs of all surrounding particles are reduced, manifesting that this resolution enhancement is universal. We have employed the technique to image many gold nanospheres and found that the averaged FWHM of a single particle is 120 ± 4 nm at this intensity.

Since the concept and setup of SUSI is similar to STED microscopy, the theoretical resolution can be analyzed accordingly by


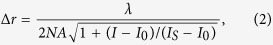


where λ is excitation wavelength, *NA* is numerical aperture, *I* is excitation intensity, *I*_*0*_ is threshold intensity when the suppression starts, and *I*_*S*_ is saturation intensity (defined as the excitation intensity when half of the scattering is suppressed)^35^,^36^. By fitting Fig. 1(a) with an exponential decay curve (green line), the values of *I*_*0*_ and *I*_*S*_ are determined to be 6.0 × 10^4^ W/cm^2^ and 1.4 × 10^5^ W/cm^2^, respectively. In Fig. 3(b), the λ = 592 nm laser intensity is 2 × 10^5^ W/cm^2^, so the expected resolution is 117 nm, agreeing very well to our experimental results.

It is known that as a coherent signal, scattering imaging cannot be described by simple convolution of PSF and sample geometry. Thus, to improve spatial resolution of scattering images, complex deconvolution, which considers both amplitude and phase, has to be incorporated^37^. However, as we show in Fig. 3(c), with the aid of simple deconvolution, the FWHM of the scattering image further decreases to 60 nm, which reaches the spatial resolution to λ/9, a remarkable result comparable to other superresolution microscopies with similar setup^38^. The line profiles of the selected particle pair are given below each image panel of Fig. 3, showing quantitative resolution enhancement of SUSI. It is interesting to notice that the FWHM after deconvolution becomes smaller than the particle diameter. The reason might be that the effective scattering center is smaller than the physical size of the particle, or that the coherence of scattering results in interference and thus reduced FWHM.

## Discussion

We have demonstrated for the first time that plasmonic scattering from individual gold nanospheres can be reversibly switched on/off by another laser beam with laser power less than 100 μW. Since the all-optical switch is based on hot lattice nonlinearity, one major issue is how fast the switching speed is. Ultrafast thermo-optical nonlinearity from hot lattice has been proposed recently^39^. The switching speed is determined by the time scale of heating up and cooling down a single nanoparticle. Theoretically, it can be as fast as a few picoseconds. However, at this temporal scale, the contribution of hot electron and hot lattice mixed up, and the former is likely to be dominant. For example, as we mentioned earlier, the ultrafast all-optical switch based on absorption from ensemble of nanoparticles has been demonstrated^22^,^23^, and the response time is only a few picoseconds, mainly from the hot electron contribution. We have also carried out analogous measurements based on a femtosecond pump-probe setup, resulting in similarly high-speed operation, but the modulation depth also drops significantly to only a few percent. As discussed earlier, in bulk gold, the nonlinearity of hot lattice from nanosecond excitation is three orders larger than that of hot electron from femtosecond excitation. Therefore, we expect the optimal excitation, and thus speed, of the photothermal all-optical switch will be on the nanosecond time scale. As shown in Fig. 3, the imaging speed is about 1 second per frame, so the pixel dwell time is less than 10 μs, and the thermal response must be much faster than this time scale. More experiments are underway to optimize the tradeoff between speed and modulation depth. In addition, with proper metamaterial designs to enhance two-photon interactions, the lifetime of nonlinear absorption/scattering may be further reduced to 100 femtoseconds or shorter^40^, thus providing an attractive possibility for all-optical ultrafast switch with ultra-small sizes.

We also demonstrated that one novel application of the plasmonic all-optical switch is to enhance spatial resolution of optical microscopy based on a STED-like technique. It will be highly desirable to further increase the spatial resolution by enhancing the ratio of scattering suppression. In our current results, only one order of magnitude suppression can be achieved. Since the mechanism is found to be thermal effect, further enhancements are possible if the thermal conduction is reduced in the ballistic thermal conduction regime, or when the nanoparticles are coated with materials of low conductivities. In addition, other nonlinearities may interfere with the effect at higher excitation intensity, as we have reported earlier^41^. More studies will be required to maximize the suppression ratio.

One major concern in all superresolution techniques is the high-intensity requirement, which may cause sample damage. To reduce the saturation intensity in conventional STED or reversible saturable optical fluorescence transitions (RESOLFT), great efforts have been put to select fluorophores with increased cross section or switching lifetime^42^,^43^. However, the imaging speed is significantly reduced after employing long-lifetime fluorophores. Because the scattering cross sections of gold nanoparticles are much larger than typical emission cross sections of fluorophores, the saturation intensity required for SUSI is expected to be much lower, while maintaining reasonable imaging speed due to its fast switching. Indeed, we have found that the saturation intensity *I*_*S*_ (~1.5 × 10^5^ W/cm^2^) required for SUSI is at least 100-fold less than those required for conventional continuous-wave STED^44^,^45^. The unique advantages of low intensity threshold while maintaining the capability of fast scanning may make SUSI a suitable platform for high-resolution, long-term imaging applications.

## Methods

### Sample preparation

The gold nanoparticles are from BBI international. The diameter measured by scanning electron microscopy is 81.5 ± 5.4 nm, corresponding well to the 80-nm specification of manufacturer. Before experiment, the nanoparticle solution was sonicated for 15 minutes to prevent aggregation. The solution were then placed on MAS-coated slide glasses (Matsunami Glass, Japan, http://www.matsunami-glass.co.jp/english/life/clinical_g/data18.html) for 15 minutes and repetitively rinsed to form a sparse distribution of fixed gold nanoparticles on the glass surface. To prevent the strong reflection from glass interfaces during optical measurement, refractive index matching oil is added around the gold nanoparticles.

### Microscope setup

**Figure 4:**
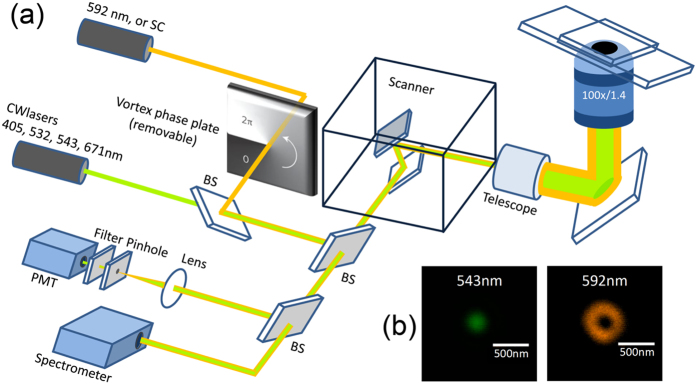
Experimental setup. (**a**) Optical construction of the multi-wavelength confocal microscope. A 592-nm beam plus a vortex phase plate is incorporated to improve resolution. SC: supercontinuum; BS: beamsplitter; PMT: photomultiplier tube. (**b**) Measured point spread function of 543 nm and 592 nm (with vortex phase plate).

Our experiment was carried out with a Leica TCS STED CW microscope system, which is designed to achieve superresolution imaging based on STED. The advantage of using continuous-wave STED is free of synchronization^45^. The configuration of the system is shown in Fig. 4(a). The system is equipped with multiple continuous-wave laser lines, including 405 nm, 532 nm, 543 nm, 561 nm, and 671 nm for confocal excitation. A 1.5-W 592-nm laser is included for fluorescence depletion purpose, and an additional supercontinuum source (Fianium, UK) is used to acquire scattering spectrum from a single nanoparticle. In our case, this strong orange laser serves the purpose of scattering suppression for 80-nm gold nanospheres since this laser wavelength falls at the peak of corresponding SPR. An oil-immersion objective (HCX PL APO 100x/1.4 Oil STED, Leica, Germany) that is optimized for STED microscopy is used for both intensity and imaging measurements. The scattering versus excitation intensity dependence is measured with these multiple laser lines under a reflection confocal detection scheme individually. For intensity measurement of the all-optical switch in Fig. 1, the weak 543-nm beam is overlapped with the strong 592-nm beam without vortex phase plate. Since both lasers are within the SPR band of the 80-nm gold nanospheres, when the intensity of 592-nm is increased, the scattering of 543-nm is expected to be suppressed.

For spectral measurement in Fig. 2, a 561-nm and a supercontinuum sources are overlapped and focused onto a single nanoparticle. The former suppresses scattering of the latter, demonstrating broadband operation. The reason to use the 561-nm laser is to allow the observation of SPR peak. In this set of experiment, linearly polarized 561-nm laser is used to study the polarization dependency of suppression. Since the dichroic beamsplitters in the microscope system might change the polarization status, to avoid the effect, pure TE and TM polarizations relative to the beamsplitters are used. The polarization linearity of laser is better than 100:1, measured at the back pupil of the objective.

To achieve superresolution imaging, a vortex phase plate is inserted into the optical path of the circularly-polarized 592-nm laser to modify its point spread function into a donut shape, as shown in Fig. 4(b). The donut-shaped 592-nm is again overlapped with the weak 543-nm, whose point spread function is still solid. It is important to precisely overlap the centers of these two beams. When the intensity of 592-nm laser is increased, the scattering of 543-nm can be suppressed in the peripheral, but since the intensity of 592-nm is diminishing in the center, the scattering of 543-nm at the central part is not affected. As a result, the FWHM of 543-nm is effectively reduced, providing enhanced resolution based on non-bleaching scattering contrast. To further improve the spatial resolution, the image was processed by deconvolution based on a commercial software (Titan, Huygens, Netherlands), which generates a theoretical PSF from the known objective parameter, and restores resolution by maximum likelihood estimation.

### Derivation of nonlinear refractive index n_2_

It is well known that the nonlinear refractive index *n*_*2*_ is used to describe a general optical Kerr effect of refractive index: *n* = *n*_0_ + *n*_2_*I*_*in*_, where *n*_*0*_ is the linear index, and *I*_*in*_ is incident intensity. The index can be separated into real part and imaginary part. The imaginary part (typically termed as *k*) corresponds to absorption coefficient *α*, while the real part (typically termed as *n*) dominates the scattering/reflection behavior. The effect of nonlinear absorption, as reported in many z-scan literatures, is quantitatively illustrated by the second-order nonlinear equation: *α* = *α*_0_ + *βI*_*in*_^46^.

Here we assume that the scattering signal comes from the real part index mismatch Δ*n* between the metallic nanoparticle and the surrounding medium^47^. From Rayleigh-Gans formalism, the scattering coefficient is *α*_*S*_ = *g*_*S*_(Δ*n*)^2^, where *g*_*S*_ is a proportional parameter that is dependent on geometry of the particles. The index mismatch is contributed by both linear Δ*n*_*L*_ and nonlinear components Δ*n*_*NL*_, i.e. (Δ*n*)^2^ = (Δ*n*_*L*_ + Δ*n*_*NL*_)^2^ ≈ (Δ*n*_*L*_)^2^ + 2Δ*n*_*L*_Δ*n*_*NL*_. By further assuming that the nonlinear index of the surrounding medium is negligible, the nonlinear index mismatch term Δ*n*_*NL*_ is equal to the nonlinear index of gold nanoparticle *n*_2_*I*_*in*_. Therefore, the scattering coefficient can be linked to nonlinear index via this equation: 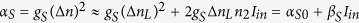 where *α*_*S*0_ and *β*_*S*_ are the linear and nonlinear scattering coefficients.

**Figure 5:**
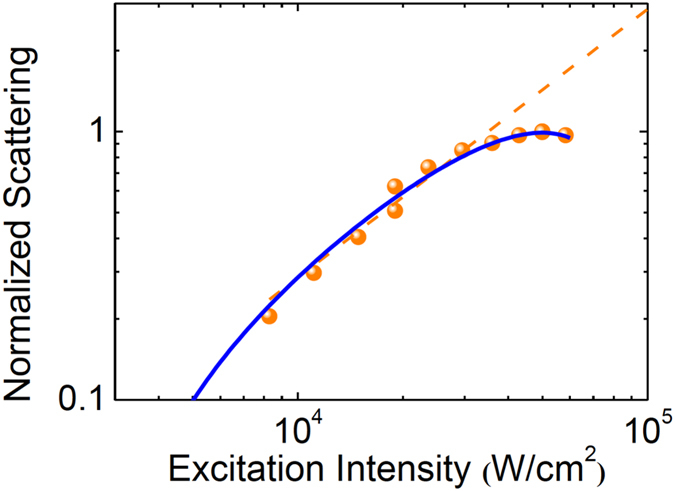
The scattering intensity dependence of 80-nm particles with 592-nm excitation. Orange circles are experimental points, orange dashed line shows the linear trend, and the blue curve is the fitting result with the quadratic equation.

From the above discussion, the scattering intensity is proportional to 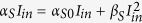. The equation can be further simplified by normalizing with the linear component, leading to the expression of scattering intensity proportional to 
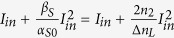
. By fitting with the nonlinear scattering response at each wavelength, the corresponding nonlinear index can be found. Fig. 5 shows the example of 592 nm, where clear nonlinear (saturable) scattering is observed^49^. At this wavelength, the real part of the gold index is about 0.27^48^, and the index of the surrounding oil is 1.52, so 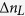 is 1.25. The second-order nonlinear term is found to be 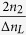 = −1.1 × 10^−9^, so the nonlinear index is *n*_2_ = −8.8 × 10^−10^ m^2^/W.

## Additional Information

**How to cite this article**: Wu, H.-Y. *et al*. Ultrasmall all-optical plasmonic switch and its application to superresolution imaging. *Sci. Rep.* **6**, 24293; doi: 10.1038/srep24293 (2016).
